# Elderhood in Protestant Religious Contexts: Stepping Stones in Religious Language

**DOI:** 10.1093/geroni/igab046.1919

**Published:** 2021-12-17

**Authors:** Jenni Spännäri

**Affiliations:** University of Eastern Finland, Joensuu, Pohjois-Karjala, Finland

## Abstract

Elderhood is an emerging concept for making meaning in older age, often contextualized in spiritual but not religious traditions. But what kinds of frameworks for elderhood are woven into protestant religious contexts? This paper explores 943 texts written by Finnish older adults in study groups organized by a pensioners’ organization. A key finding is that religious language – known through religious songs and prayers learned by heart at school – offers a medium to explore and express their elderhood. The writers creatively use the rhythm and wordings of these textual patterns to position themselves as a group of older persons with a special contribution to make to society. These results will aid examining elderhood and its potential in various contexts where the concept might not be explicitly used. This examination potentially leads to new ways to support experiences of elderhood and thus to offer an alternative view to countering ageism.

